# *Lactococcus garvieae*, an unusual pathogen in infective endocarditis: case report and review of the literature

**DOI:** 10.1186/s12879-019-3912-8

**Published:** 2019-04-03

**Authors:** Alexandre Malek, Alejandro De la Hoz, Sara Isabel Gomez-Villegas, Cima Nowbakht, Cesar A. Arias

**Affiliations:** 1 Department of Internal Medicine, Division of Infectious Diseases, UTHealth - McGovern Medical School, Houston, TX USA; 2 Grupo de Investigación en Enfermedades Infecciosas, Hospital Universitario San Ignacio, Pontificia Universidad Javeriana, Bogotá, Colombia; 3 Universidad de Antioquia, School of Medicine, Medellin, Colombia; 4 Center for Antimicrobial Resistance and Microbial Genomics (CARMiG), UTHealth - McGovern Medical School, Houston, TX USA; 5 Center for Infectious Diseases, UTHealth – School of Public Health, Houston, TX USA; 6 Molecular Genetics and Antimicrobial Resistance Unit and International Center for Microbial Genomics, Universidad El Bosque, Bogota, Colombia; 7 University of Texas Health Science Center at Houston (UTHealth), 6431 Fannin St. MSB 2.112, Houston, TX 77030 USA

**Keywords:** Lactococcus, Case report, Gram positive cocci, Infective endocarditis, Diagnosis, Treatment, Risk factors

## Abstract

**Background:**

*Lactococcus garvieae* is an unusual cause of infective endocarditis (IE). No current diagnostic and therapeutic guidelines are available to treat IE caused by these organisms. Based on a case report, we provide a review of the literature of IE caused by *L. garvieae* and highlight diagnostic and treatment challenges of these infections and implications for management.

**Case presentation:**

A 50-year-old Asian male with mitral prosthetic valve presented to the hospital with intracranial haemorrhage, which was successfully treated. Three weeks later, he complained of generalized malaise. Further work up revealed blood cultures positive for Gram-positive cocci identified as *L. garvieae* by MALDI-TOF. An echocardiogram confirmed the diagnosis of IE. Susceptibility testing showed resistance only to clindamycin. Vancomycin plus gentamicin were started as empirical therapy and, subsequently, the combination of ceftriaxone plus gentamicin was used after susceptibility studies were available. After two weeks of combination therapy, ceftriaxone was continued as monotherapy for six additional weeks with good outcome.

**Conclusions:**

Twenty-five cases of IE by *Lactococcus garvieae* have been reported in the literature. Compared to other Gram-positive cocci, *L. garvieae* affects more frequently patients with prosthetic valves. IE presents in a subacute manner and the case fatality rate can be as high as 16%, comparable to that of streptococcal IE (15.7%). Reliable methods for identification of *L. garvieae* include MALDI-TOF, 16S RNA PCR, API 32 strep kit and BD Automated Phoenix System. Recommended antimicrobials for *L. garvieae* IE are ampicillin, amoxicillin, ceftriaxone or vancomycin in monotherapy or in combination with gentamicin.

## Background

*Lactococcus garvieae* are Gram-positive cocci previously considered part of the genus *Streptococcus*. In 1985, these organisms were classified within the genus lactococci due to DNA-DNA hybridization studies and fatty acid profiles [1–3]. Currently, the genus *Lactococcus* contains 11 species [4]. *L. garvieae* is associated with fish infections in warm water causing outbreaks of haemorrhagic sepsis in rainbow trout [2, 5]. These organisms have also been isolated from raw cow milk, goat cheese, fish, beef meat, poultry and pork meat [6]. Human infections caused by *L. garvieae* have been reported in different countries and have been associated to ingestion of raw seafood. Indeed, a study by Wang et al. found that among four patients with invasive *L. garvieae* infection, three had ingested sea food contaminated by these organisms [7]. Infective endocarditis (IE) is a known disease caused by *L. garvieae*, however, the true incidence of disease is difficult to assess since misidentification with other Gram-positive cocci like *Enterococcus* spp*.* and streptococci (employing different automatized diagnostic tools) has commonly been reported [8, 9]. Here, we report a case of *L. garvieae* IE and describe the risk factors associated with this disease, the diagnostic challenges to identify these organisms and therapeutic approaches used to treat these infections. We seek to provide clinicians with relevant and updated information on the diagnosis and management of IE caused by the genus *L. garvieae.*

We searched MEDLINE, EMBASE and LILACS using the following MeSH, major and free terms: “endocarditis”, “endocarditis, bacterial”, “endocarditis, subacute bacterial”, “endocarditis bacteriana”, “endocarditis bacteriana subaguda” and “lactococo”, “lactococcus”, “lactococcus lactis”, “lactococcus garvieae”, “lactococcus garvieae endocarditis”. We selected all the articles in Spanish, English and French published before March 2018 that included case reports of endocarditis and *Lactococcus* in the titles.

### Case presentation

A 50-year-old Asian man with history of rheumatic heart disease (without hypertension) and mechanical prosthetic mitral valve replacement 5 years before admission, dyslipidaemia and reflux esophagitis presented to the emergency room with severe bilateral occipital headache. He was diagnosed with an intracranial haemorrhage confirmed by CT brain. At the time of admission, his INR was within therapeutic range (2.35). After initial work up, the patient was hospitalized for 10 days and discharged without any residual neurologic sequelae. Atorvastatin was prescribed. No fever or elevation of the C reactive protein (CRP) or erythrocyte sedimentation rate (ESR) were identified during the admission. He worked as an accountant and had been living in the US for the past 30 years with no recent travel outside the US. Three weeks later, he complained of flu-like symptoms and oseltamivir was prescribed. A week later, the patient returned to the hospital with epistaxis, haematuria, and malaise without fever. Physical examination was unremarkable with normal neurologic exam, except for a pansystolic heart murmur. Blood tests showed elevated white blood count (14.5 × 10^9^/L) and serum creatinine of 1.54 mg/dl (Normal value: 0.8–1.2 mg/dl). CRP and ESR were also elevated (34.5 mg/dl and 75 mm/h, respectively). A Chest X ray was found without acute abnormalities and the urine analysis showed no abnormalities. Three days after admission, blood cultures were positive for Gram-positive cocci in chains in 4 out of 4 bottles. Transthoracic echocardiography was inconclusive, but a transoesophageal echocardiography (TEE) revealed a 0.8 cm vegetation on the ventricular side of the native aortic valve without valve dysfunction, confirming the diagnosis of IE. Empirical intravenous antibiotic therapy was started with vancomycin 30 mg/kg/day in divided doses and gentamicin 3 mg/kg/day. The organism was recovered on blood agar and was identified by MALDI-TOF as *Lactococcus garvieae*. Susceptibility testing showed resistance to clindamycin, whereas it was susceptible to penicillin (MIC 0.25 μg/ml), ceftriaxone (MIC 0.25 μg/ml), vancomycin (MIC 1.5 μg/ml) and levofloxacin (MIC 2 μg/ml). With these results, vancomycin was switched to ceftriaxone 2 g IV twice daily plus gentamicin as combination therapy for the first 2 weeks. This regimen was chosen based on previous cases since no specific guidelines exist on how to treat these organisms. Gentamicin was stopped after two weeks and ceftriaxone was continued for 4 additional weeks pending a surgical decision. In the setting of intracranial bleed and IE, rupture of a mycotic aneurysm was highly suspected and the patient was considered a possible surgical candidate for aortic valve replacement. CT angiography of the brain (5 weeks after the initial episode of intracranial bleed) showed encephalomalacia in the left parietal and occipital lobes with subacute to chronic haemorrhage, with no mycotic aneurysms. After several discussions, the stroke team agreed on resuming anticoagulation with heparin IV drip (considering that the patient had a “chronic” bleed without active haemorrhage and that the risk of embolism was high due to the presence of a mechanical heart valve and IE). It was also suggested postponing aortic valve replacement for at least 4 weeks after effective antimicrobial therapy. After 4 weeks of therapy, decrease of inflammatory markers (CRP to 8.5 mg/dl and ESR to 40 mm/h) was observed and repeat blood cultures were negative.

Upon further questioning, the patient admitted that his diet was rich in grilled fish. Additionally, he reported a long history of chronic epigastric pain for 5 years, for which he had been taking over the counter medicines. An esophagogastroduodenoscopy showed severe gastritis and reflux esophagitis. After 6 weeks of treatment for IE, the patient had clinical improvement with no recurrence of infection but repeat TEE revealed severe aortic valve insufficiency. He underwent mechanical aortic valve replacement without complications and cultures from the excised valve were sterile.

## Discussion and conclusion

Different clinical presentations of subacute IE make it challenging to make an early diagnosis of infection and can cause delays in appropriate treatment. In our case, treatment of IE was delayed due to low suspicion of the disease at presentation and the occurrence of the intracranial haemorrhage. Importantly, collection of blood cultures as soon as infection was suspected, led to isolation of *L. garvieae* and identification using MALDI-TOF.

A total of 25 cases of IE caused by *L. garvieae* were identified in the literature review [8–30]. Among the 25 cases of IE caused by *L. garvieae* (Table 1), 58% were reported in men and the median age of presentation was 68 years. Median duration of symptoms before consulting was 14 days (IQR = 6.2–21). The most common reported symptoms were fever (68%) and chills (28%). Presence of heart murmurs was the most common finding in the physical examination (72%). Laboratory tests usually showed leucocytosis, elevated CRP and ESR. Echocardiogram was reported in 24 out of 25 cases and vegetations were identified in 83.3%. The mitral valve was the most frequently involved valve. Colonoscopy was performed in 5 cases, all of which reported colonic polyps. The median duration of antimicrobial therapy was 42 days (IQR 41–45.5).

**Table 1:** Clinical, microbiological and management characteristics of IE by *L. garvieae*

Reference	Country	Age	Sex	Associated factors for IE by *L. garvieae* and comorbidities	Symptoms and duration (days)	Physical examination	Laboratories and Echocardiography (Vegetation size)	Identification	Susceptibility	Therapy and days of treatment	Complications and Outcome
Clavero et al. 2017	Chile	72	F	AV fistulas, Diverticulosis, CKD, DM 2 and HTN, colonic polyps	Chills, fever (1)	Fever, systolic murmur, pulmonary crackles	TEE: mitral vegetation (4 mm) Labs: Leucocytosis elevated CRP Colonoscopy: Colonic polyps	LG: Vitek 2^¥^, MS and 16S rRNA PCR.	MIC: VAN: 2 μg/ml, CTX 0.25 µg/ml PEN: 0.5 µg/ml. Kirby Bauer: Sensitive: ERY, CIP, SXT, AMC Resistant: CLI. Applying the criteria for B-haemolytic streptococci	Empiric: CLO + AMK Directed: VAN + GEN µg (NI)	Shock, respiratory failure (Died)
Lim and Jenkins 2017	UK	57	M	Cooked fish, gallstones, renal stones, Colonic polyps	Fever weight loss (60)	Pansystolic murmur	TEE: mitral valve vegetation (NI) and regurgitation. Colonoscopy: Duodenal polyps	LG: MS	PEN MIC: 1 mg/L Etest BioMérieux, S: GEN 200 µg Oxoid by diffusion disc testing.	AMX + GEN (42d) Valve replacement	No complications, (Alive)
Landeloos et al. 2017	Belgium	82	F	Prosthetic mitral valve, previous endocarditis, Cooked fish, colonic polyps, FA, HTN, Osteoporosis.	Fever, hyporexia, dyspnoea (14)	Bradycardia, Basal lung crepitations, reinforce caused S2	TEE: mitral vegetation (10x5mm) Labs: Elevated CRP no leucocytosis. Colonoscopy: Colonic polyps.	LG: MS, 16S rRNA PCR.	NI	Empiric: CRO Directed: MXF Changed: PEN + GEN Ambulatory: AMX monotherapy (42d) No valve replacement	No complications (Alive)
Bazemore et al. 2016	USA	45	M	Multiple substance abuse. Repair of aortic root aneurysm, Hepatitis C and cirrhosis	Malaise, weakness (60)	Fever, systolic murmur	No Echo reported. Leucocytosis, Elevated CRP and ESR.	LG: MS	E-test: sensitive to CRO and VAN Cut-off values for *S. bovis*	Empiric: TZP + VAN Directed: CRO + GEN (NI) + Valve repair	Aortic valve dehiscence (Alive)
Suh et al. 2016	South Korea	75	F	Mitral valve prosthesis, eats sea fresh food.	Dyspnoea (3)	Holosystolic murmur	TTE: mitral vegetation (16 mm) Labs: leucocytosis, Elevated CRP	LG: Vitek 2^¥^, 16S rRNA PCR.	MicroScan MICroSTREP plus panel: PEN 0.12 µg/ml, AMC: 0.5/0.25 µg/ml, CRO 0.25 µg/ml, CTX 0.25 µg/ml, MEM 0.06 µg/ml, VAN 1 µg/ml, LVX 1 µg/ml, CLI > 0.5 mcg/ml (only R to CLI). E test (bioMérieux, Marcy lEtoile, France) PEN 0.75 mg/L, CRO 0.38 mg/L, TEC 0.125 mg/L. Susceptibility CLSI for viridans streptococci.	Empiric: CRO + GEN + RIF Directed: TEC Changed: CRO monotherapy (40d) Aortic and mitral replacement	Heart failure (Alive)
Heras Cañas et al. 2015	Spain	68	M	Prosthetic aortic valve, HTN, dyslipidaemia, Hodgkin lymphoma in remission, AV block.	Fever, Dyspnoea (10)	NI	TTE: mitral vegetations (NI). Laboratories: Leucocytosis, elevate CRP.	LG: MS and 16S rRNA PCR	Streptococci breakpoints: S: CTX: 0.38 µg/ml, ERY: 0.25 mg/dl, VAN 1 µg/ml, LVX 1.5 µg/ml, VAN, AMP, CTX, OXA. I: PEN-I MIC 0.75 µg/ml, R: CLI 1 µg/ml.	Empiric: DAP + AMP + CRO Directed: DAP+AMP + CRO + GEN (NI) + Valve replacement	AKI, Aortic valve dehiscence (Alive)
Igneri et al. 2015	USA	83	M	Prosthetic aortic valve, Recent dental intervention, CHF, CLL, Prostate cancer, Coarctation of aorta, CABG	Malaise, fever, vomit, headache, cough, myalgia, diaphoresis (7)	NI	TTE: Could not exclude vegetations, Labs: Leucocytosis, Elevated CRP	NI	NI	Empiric: AMP + GEN Directed: CTX + GEN (42d) Valve surgery	No complication, (Alive)
Ortiz et al. 2014	Spain	70	F	No risk factors or comorbidities	Dyspnoea (NI)	Holosystolic murmur, fever	TTE: mitral vegetation (NI) Labs: Leucocytosis and elevated CRP	NI	S: CTX, CIP, ERY, DAP, VAN.	Empiric: AMC + GEN Directed: VAN monotherapy (42d) valve surgery	No complications (Alive)
	Spain	77	F	Colorectal cancer, HTN, CLL	Back pain, fever (2)	Purpuric lesions in extremities, fever	TEE: mitral and aortic vegetation (NI) Labs: NI	NI	S: PEN, AMC, CIP, VAN.	AMP + GEN (NI) No valve surgery	AKI, Heart failure (Died)
Tsur et al. 2014	Israel	76	M	Raw fish, Prosthetic aortic valve, CHF, AF, DM 2, HTN, Oesophageal carcinoma.	Constipation, fever (NI)	Fever, Tachycardia, Systolic murmur.	TEE: Vegetation biologic prosthetic valve (NI) Labs: Leucocytosis	Lactococcus: API 32 strep kit^a^, 16S rRNA PCR	S: CRO and GEN. I: PEN R: CLI	Empiric: CRO Directed: CRO+ GEN (NI) No valve replacement	No complications (Alive)
Rasmussen et al. 2014	Sweden	81	M	Prosthetic aortic valve, rectal diverticulosis, CAD, CABG, AF	Malaise, headache, dysphasia (NI)	Fever, systolic murmur.	TEE: vegetations mitral valve and prosthetic valve (NI) Labs: Elevated CRP	LG: Vitek 2^¥^, 16S rRNA PCR	PEN: 0.5 mg/L, TOB: 2 mg/L.	PEN + TOB (28d) No valve replacement	Subdural hematoma (Alive)
Navas et al. 2013	USA	64	M	Previous mitral valve repair, Dental intervention, CAD, Cardiac defibrillator, DM2, COPD	Fatigue, weight loss, hyporexia, weakness, fever (NI)	NI	Echo not specified: Aortic vegetations	LG: Vitek 2^¥^, 16S rRNA PCR and MS Wrong ID: Microscan^b^	Streptococcus breakpoints: I: PEN and AMP R: CLI	VAN monotherapy (42d) Aortic valve replacement. Removal of pacemaker	Intracardiac device infection (Alive)
Fleming et al. 2012	USA	68	M	Prosthetic aortic valve, NHL in remission. Colonic polyps	Migratory arthralgias, dyspnoea, hyporexia, fatigue, weight loss, fever (21)	Systolic ejection murmur. Splinter haemorrhage in nails of left hand	Echo not specified: Vegetation mitral valve. (NI) Labs: Elevated CRP and ESR Colonoscopy: Colonic polyps	LG: Vitek 2^¥^, 16S rRNA PCR	Breakpoints for VGS: S: VAN, SAM, CRO, TZP R: AMP, GEN, CLI.	Empiric: AMP + GEN Directed: VAN monotherapy (42d) No valve replacement	No information of complications (Died)
Russo et al. 2012	Italy	63	M	Ascending aorta and aortic valve replacement, previous endocarditis, HTN.	Fever, chills, Pharyngodini, weakness. (7)	Systolic murmur, hepatomegaly	TEE: mitral vegetation (NI) Labs: Elevated CRP	LG: API 32^b^, Vitek 2^¥^, 16S rRNA PCR	EUCAST Streptococci breakpoint: S: ERY/S: 0.125 μg/ml, CTX 0.5 μg/ml LVX: 0.5 μg/ml, AMP: 0.25 μg/ml, AMC: 0.5 μg/ml, CIP: 0.75 μg/ml, DAP: 0.125 μg/ml, GEN: 2 μg/ml VAN: 2 μg/ml TEC: 0.5 μg/ml I: PEN: 2 μg/ml R: CLI: > 64 μg/ml, RIF > 64 µg/ml	Empiric: VAN + GEN Directed: AMP monotherapy (14d) No valve replacement	No complications (Alive)
Watanabe et al. 2011	Japan	55	F	No risk factors or comorbidities	Malaise, myalgia, fever (60)	Systolic murmur, painful black induration in finger.	TEE: Mitral vegetation (10 mm) Labs: No leucocytosis Elevated CRP	LG: Rapid ID32 Strep. ^a^, 16S rRNA	E test: (AB Biodisk, Dalvagen, Solna, Sweden): ERY 0.25 mg/L, CLI: > 256, VAN: 0.38 mg/L, LZD: 2 mg/L, PEN 0.5 mg/L, CRO 0.38 1mg/L, GEN: 1.5 mg/L, STR: 64 mg/L	Empiric: PEN + GEN. Directed: CRO + GEN (63d) No valve surgery	Septic embolism, stroke, aspirative pneumonia (Alive)
Zuily et al. 2011	France	64	F	Mitral valve prosthesis, fresh seafood, Pacemaker, Hepatitis C cirrhosis,	Fever (NI)	Fever, Murmur.	TEE: Mitral vegetations (NI) Labs: Elevated CRP and ESR. Leucocytosis	LG: PCR	NI	AMX + GEN (42d) no surgery	No complications (Alive)
Wilbring et al. 2011	Germany	55	M	Fish farmer, mechanical tricuspid valve prosthesis, periodontitis	Chills, fever, dyspnoea (14)	Murmur	TEE: Mechanical prosthetic valve vegetation (7x9mm) Labs: Leucocytosis and elevated CRP	NI	NI	Inpatient: GEN + VAN + RIF ambulatory: LVX + AMC (56d) No valve replacement	No complications (Alive)
Hirakawa et al. 2011	Brazil	58	F	Mitral prosthetic valve, fish and cheese often. Dental prosthesis and recent gingival perforation. DM 2, HTN, Dyslipidaemia	Fever, chills, diaphoresis, erythematous nodules in hands and legs, myalgia, weakness. (6)	Fever, Osler nodes on left hand and legs.	TEE: No vegetations Labs: Elevated CRP and ESR no leucocytosis.	LG: Not specified biochemical tests. PCR.	S: PEN, GEN, VAN. R CLI	VAN monotherapy (28d) No valve surgery	No complications (Alive)
Li et al. 2008	Taiwan	41	M	No risk factors or comorbidities	Slurred speech (1)	Right hemiplegia loss of right body sensation, right positive Babinski sign, murmur, fever	TEE: Mitral vegetation and rupture of chordae tendineae (NI) Labs: Leucocytosis and elevated CRP	LG: Vitek 2^¥^, Automated Pheonix^c^, 16S rRNA PCR^e^	I: PEN: 0.75 µg/ml.	PEN + GEN (30d) Valve replacement	Septic emboli, stroke, shock (Alive)
Yiu et al. 2007	China	67	M	Heart rheumatic disease, previous endocarditis, eats fresh fish, AF	Chills, fever (21)	Fever, mitral regurgitation murmur	TEE: Mitral vegetation (10x1mm) Labs: Elevated ESR Neutrophilia	NI	NI	AMP monotherapy (42d) Valve replacement	Partial rupture of mitral valve (Alive)
Wang et al. 2006	Taiwan	72	M	Kidney stones, mitral valve prolapse. Raw fish consumption, gastric ulcer	Fever, purpuric leg lesions (14)	Systolic murmur.	Echo not specified: Severe mitral regurgitation, prolapse of posterior mitral valve, echogenic mass on the posterior mitral valve Labs: No leucocytosis. Endoscopy: Gastric ulcer	LG: ID32 STREP; BioMérieux, Hazelwood, MO, USA, 16S rRNA PCR	NI	PEN + GEN (42d)	No complications (Alive)
Vinh et al. 2006	Canada	80	M	DM2, Hyperlipidaemia, CAD, CHF	Dyspnoea, epigastric discomfort. (NI)	Midsystolic murmur	TEE: Aortic vegetations (24 mm) Labs: NI	Wrong ID: API 20^d^ (L. lactis), Vitek 2^¥^(Enterococcus), 16S rRNA PCR	CLSI *Enterococcus spp*. breakpoints: S: PEN, CIP, OFX, LVX, TET, VAN. CLSI *Streptococcus spp.* breakpoints: S: AMP, VAN, GEN I: PEN R: CLI	Empiric inpatient: AMP Ambulatory: PEN and then switched to AMP again. Monotherapy (56d) Valve replacement	No complications (Alive)
Fihman et al. 2005	France	86	M	Prosthetic aortic valve, Cholecystectomy.	Fever, right hip pain (21)	Fever, respiratory distress	TEE: Aortic vegetation (10 mm) Labs: Leucocytosis and elevated CRP.	LG: API 32^a^, 16S rRNA PCR	E test: MIC: PEN: 0.75 μg/ml, AMX: 0.5 μg/ml CTX: 0.38 μg/ml, VAN: 1.5 μg/ml, TEC: 0.38 μg/ml, CLI >8 µg/ml	Inpatient: AMX + GEN Ambulatory: AMX monotherapy (49d) Valve repair	No complications (Alive)
James et al. 2000	UK	56	F	Aortic valve prosthesis	Low back pain, chills, night sweat, weight loss, hyporexia (63)	Low back tenderness, splinter haemorrhages In nails, murmur	TTE: No vegetations Labs: Elevated ESR and CRP. No leucocytosis	LG: API Strep ^f^	With streptococci reference laboratory (Respiratory and systemic reference laboratory London UK).	Empiric: VAN Directed: TEC monotherapy (56d)	Osteomyelitis (Alive)
Fefer et al. 1998	USA	84	F	Pacemaker for heart block, Aortic valve prosthesis, omeprazole, hypertrophic cardiomyopathy, ITP, hypothyroidism.	Hyporexia, weakness, dyspnoea (NI)	Holosystolic murmur, bilateral pulmonary rales	TEE: Ruptured chordae tendineae. Labs: Leucocytosis. Negative colonoscopy.	LG: Biochemical tests.	NCCLS Staphylococcus spp. breakpoints: S: VAN, AMP, CRO	Empiric: AMP + GEN Directed: CRO monotherapy (NI)	Intracranial haemorrhage, Rupture of chordae tendineae (Died)

*NI* No information, *AMK* amikacin, *AMX* amoxicillin, *AMC* amoxicillin-clavulanic acid, *AMP* ampicillin, *CFZ* cefazolin, *CDN* cefditoren, *CTX* cefotaxime, *CRO* ceftriaxone, *CEF* cephalothin, *CHL* chloramphenicol, *CIP* ciprofloxacin, *CLR* clarithromycin, *CLI* clindamycin, *DAP* daptomycin, *ERY* erythromycin, *GEN* gentamicin, *LVX* levofloxacin, *LZD* linezolid, *MEM* meropenem, *MXF* moxifloxacin, *OFX* ofloxacin, *PEN* penicillin, *TZP* piperacillin-tazobactam, *RIF* rifampin, *STR* streptomycin, *TEC* teicoplanin, *TET* tetracycline, *TOB* tobramycin, *SXT* trimethoprim-sulfamethoxazole, *VAN* vancomycin, *MIC* Minimal inhibitory concentration, *S* Sensitive, *I* Intermediate, *R* resistant, *VGS* Viridans Group Streptococci, *CKD* Chronic kidney disease, *AKI* Acute kidney injury, *DM2* Diabetes mellitus type 2, *AF* Atrial fibrillation, *CHF* Cardiac heart failure, *NHL* Non-Hodgkin Lymphoma, *COPD* Chronic obstructive pulmonary disease, *CLL* Chronic lymphocytic leukaemia, *CAD* Coronary artery disease, *CABG* Coronary artery bypass graft, *LG* Lactococcus garvieae a Manual API 32 strep kit, automated Vitek 2 kit with GP identification card (BioMérieux Marcy l’etoile, france), b Microscan walk away system (dade behring, inc., Sacramento, CA), c:Automated Phoenix system (Becton Dickinson Diagnostic systems, Franklin Lakes, NJ), d: API 20strep kit (BioMérieux), e: 500 16S ribosomal rRNA bacterial sequencing kit (PE applied Biosystems, Foster city, CA, USA) ABI PRISM 310 Genetic Analyzer (PE applied biosystems), f: API Strep (BioMérieux, Basngstoke, Hants, UK)

When compared to other Gram-positive microorganisms, *L. garvieae* seems to affect more frequently patients with prosthetic valves. In our review, 52% (*n* = 13) patients with *L garvieae* IE had prosthetic valves, while large cohorts of endocarditis caused by *Enterococcus* spp., *Streptococcus* spp., Coagulase negative Staphylococci (CoNS) and *S. aureus*, report prosthetic valve involvement in 15.3–35%, 16.3–17.2%, 28–32.2% and 15.3–16% of cases, respectively [31–33]. Complications of IE such as valve dehiscence or rupture, septic emboli, renal failure, shock, stroke and heart failure were reported in 50% (*n* = 12) of cases. Surgery for valve repair or replacement was performed in 48% of cases. The case fatality rate of *L. garvieae* IE was 16% (n: 4), which is low compared to that of other GPC such as *S. aureus* (44.4%), *Enterococcus* spp. (23%) and CoNS (33.4%), but comparable to that of streptococci IE (15.7%) [32].

The ingestion of raw sea food or exposure to fish, the presence of colonic polyps and the repeated exposure to dairy products have been postulated to be risk factors for infection by *L. garvieae* [7]. Less than half of patients with IE caused by *L. garvieae* reported ingestion of fish (including raw or cooked) [7, 15, 19, 23, 24, 26–28, 30] or were diagnosed with a concomitant GI disorder [10, 13, 19–21, 24, 28–30]. Our patient reported both conditions. The most important predisposing factor in these patients appears to be the presence of previous valvular disease. Of note, colonoscopy may be considered in patients with *L. garvieae* IE to rule out colonic polyps.

For species identification, MALDI-TOF, 16S RNA PCR, API 32 strep kit (BioMérieux, Marcy l’Etoile, France), Vitek 2 kit with GP identification card (BioMérieux) and BD Automated Phoenix System seem to be reliable techniques for the identification of *L. garvieae* in our series. However, the Vitek 2 reported misidentification of *Enterococcus spp*. as *L. garvieae* in one case [8]. In contrast, the API 20 Strep (BioMérieux, Marcy-l’Etoile, France) and Microscan walk away system (Dade Behring, inc., Sacramento, CA) often misidentified the genus *L. garvieae* [8, 9]. Since the therapeutic approach for enterococci may be different to that used for *Lactococcus*, confirmation of identification should be performed with a reliable method. As no breakpoints for antibiotic susceptibility have been determined for *Lactococcus spp.* by the CLSI or EUCAST, most authors used those for viridans-group streptococci (VGS), group B streptococci, *Enterococcus* spp. or *Staphylococcus* spp. With these breakpoints, most *L. garvieae* isolates show intermediate resistance to penicillin and resistance to clindamycin [8, 9, 13, 22].

In summary, IE caused by *L*. garvieae may be a life-threatening infection. The most important predisposing factor is previous valvular disease. An association with gastrointestinal disease and consumption of fish has been established. Reliable methods for identification of *L. garvieae* include MALDI-TOF, 16S RNA PCR, API 32 strep kit (BioMérieux, Marcy l’Etoile, France) and BD Automated Phoenix System. Based on prior case reports and our own patient case, the recommended antimicrobials for *L. garvieae* are ampicillin (2 g every 4 h), amoxicillin (200 mg/kg/day divided in 4–6 doses), ceftriaxone (2 g every 12–24 h) or vancomycin (30 mg/kg/day divided in 2–3 doses) as monotherapy or in combination with gentamicin (3 mg/kg/day). Doses were defined using the recommendations for the treatment of VGS and enterococcal IE published by the American Heart Association/Infectious Diseases Society of America (AHA-IDSA) and European Society of Cardiology guidelines [34, 35]. It is unclear if combination therapy is needed (in cases where aminoglycoside toxicity is an issue), given that 5 out of 25 patients with *L. garvieae* IE were treated with vancomycin [9, 15, 17], teicoplanin [17] or ampicillin [8] monotherapy with good outcomes. Further, the majority of patients in the *L. garvieae* group who died were treated at some point with monotherapy and combination therapy.

## Abbreviations

AF: Atrial fibrillation
AKI: Acute kidney injury,
AMC: Amoxicillin-clavulanic acid
AMK: Amikacin
AMP: Ampicillin
AMX: Amoxicillin
BD: Becton Dickinson
CABG: Coronary artery bypass graft
CAD: Coronary artery disease
CDN: Cefditoren
CEF: Cephalothin
CFZ: Cefazolin
CHF: Cardiac heart failure
CHL: Chloramphenicol
CIP: Ciprofloxacin
CKD: Chronic kidney disease
CLI: Clindamycin
CLL: Chronic lymphocytic leukaemia
CLR: Clarithromycin
CLSI: Clinical and laboratory standards institute
CLSI: Clinical and Laboratory Standards Institute
CONS: Coagulase negative staphylococci
COPD: Chronic obstructive pulmonary disease
CRO: Ceftriaxone
CRP: C Reactive Protein
CT: Computed Tomography
CTX: Cefotaxime
DAP: Daptomycin
DM2: Diabetes mellitus type 2
DNA: Deoxyribonucleic acid
ERY: Erythromycin
ESR: Erythrocyte Sedimentation Rate
EUCAST: European Committee on Antimicrobial Susceptibility Testing
GEN: Gentamicin
GI: Gastrointestinal
GP: Gram positive
GPC: Gram positive cocci
I: Intermediate
IE: Infective Endocarditis
INR: International Normalized Ratio
IQR: Interquartile range
IV: Intravenous
LG: Lactococcus garvieae
LVX: Levofloxacin
LZD: Linezolid
MALDI-TOF: Matrix-Assisted Laser Desorption/Ionization Time of Flight
MEM: Meropenem
MIC: Minimum Inhibitory Concentration
MXF: Moxifloxacin
NHL: Non-Hodgkin Lymphoma
NI: No information
OFX: Ofloxacin
PCR: Polymerase Chain Reaction
PEN: Penicillin
R: Resistant
RIF: Rifampin
RNA: Ribonucleic acid
S: Sensitive
STR: Streptomycin
SXT: Trimethoprim-sulfamethoxazole
TEC: Teicoplanin
TEE: Transoesophageal Echocardiography
TET: Tetracyclin
TOB: Tobramycin
TZP: Piperacillin-tazobactam
VAN: Vancomycin
